# TNF-α augments RANKL-dependent intestinal M cell differentiation in enteroid cultures

**DOI:** 10.1152/ajpcell.00108.2016

**Published:** 2016-07-13

**Authors:** Megan B. Wood, Daniel Rios, Ifor R. Williams

**Affiliations:** Department of Pathology and Laboratory Medicine, Emory University School of Medicine, Atlanta, Georgia

**Keywords:** M cells, enteroid culture, RANKL, TNF-α, Spi-B

## Abstract

Microfold (M) cells are phagocytic intestinal epithelial cells in the follicle-associated epithelium of Peyer's patches that transport particulate antigens from the gut lumen into the subepithelial dome. Differentiation of M cells from epithelial stem cells in intestinal crypts requires the cytokine receptor activator of NF-κB ligand (RANKL) and the transcription factor Spi-B. We used three-dimensional enteroid cultures established with small intestinal crypts from mice as a model system to investigate signaling pathways involved in M cell differentiation and the influence of other cytokines on RANKL-induced M cell differentiation. Addition of RANKL to enteroids induced expression of multiple M cell-associated genes, including *Spib*, *Ccl9* [chemokine (C-C motif) ligand 9], *Tnfaip2* (TNF-α-induced protein 2), *Anxa5* (annexin A5), and *Marcksl1* (myristoylated alanine-rich protein kinase C substrate) in 1 day. The mature M cell marker glycoprotein 2 (*Gp2*) was strongly induced by 3 days and expressed by 11% of cells in enteroids. The noncanonical NF-κB pathway was required for RANKL-induced M cell differentiation in enteroids, as addition of RANKL to enteroids from mice with a null mutation in the mitogen-activated protein kinase kinase kinase 14 (*Map3k14*) gene encoding NF-κB-inducing kinase failed to induce M cell-associated genes. While the cytokine TNF-α alone had little, if any, effect on expression of M cell-associated genes, addition of TNF-α to RANKL consistently resulted in three- to sixfold higher levels of multiple M cell-associated genes than RANKL alone. One contributing mechanism is the rapid induction by TNF-α of *Relb* and *Nfkb2* (NF-κB subunit 2), genes encoding the two subunits of the noncanonical NF-κB heterodimer. We conclude that endogenous activators of canonical NF-κB signaling present in the gut-associated lymphoid tissue microenvironment, including TNF-α, can play a supportive role in the RANKL-dependent differentiation of M cells in the follicle-associated epithelium.

microfold (M) cells are specialized epithelial cells found in the follicle-associated epithelium (FAE) of Peyer's patches (PPs). M cells are responsible for the highly efficient uptake of particulate antigens into gut-associated lymphoid tissue structures, such as PPs and isolated lymphoid follicles, that serve as inductive sites for mucosal immunity in the intestine (20). Mature M cells in the FAE are defined by unique morphological features, including blunted microvilli and an intraepithelial pocket, their capacity for efficient uptake of particulate antigens, and expression of a set of genes that distinguish M cells from neighboring FAE enterocytes and the other types of specialized enterocytes found in villous intestinal epithelium. M cells develop from Lgr5^+^ stem cells present in crypts surrounding the FAE (11). Differentiation of precursor cells into the M cell lineage requires receptor activator of NF-κB (RANK) ligand (RANKL) signaling through the RANK receptor (26) followed by induction of the Ets transcription factor Spi-B, which is restricted to the M cell lineage among enterocytes and required for full differentiation of M cells and acquisition of markers found on mature M cells such as glycoprotein 2 (GP2) (23). Not all M cell-associated markers require Spi-B expression: the selective expression of *Marcksl1* (myristoylated alanine-rich protein kinase C substrate) and *Anxa5* (annexin A5) by M cells is independent of Spi-B (23, 43). Mice with conditional deletion of the *Tnfrsf11a* (TNF-α receptor superfamily member 11A) gene encoding RANK in the intestinal epithelium have a phenotype characterized by the absence of intestinal M cells, reduced formation of germinal centers in PPs, and substantial impairment in development of a secretory IgA response after weaning (40). The scarcity of M cells within the entire intestinal epithelium has consistently presented a formidable obstacle to the development of in vitro approaches to study M cell differentiation and function.

An in vitro model system that has been used widely to study M cell biology is coculture of the human Caco-2 colonic adenocarcinoma cell line with a source of B lymphocytes in polarized Transwell cultures (24). In the presence of B cells, a subset of the Caco-2 cells exhibits enhanced transcytosis of particulate antigens that resembles one of the main phenotypic features of natural M cells in the FAE. In the original version of this coculture model of M cell-like cells, freshly isolated mouse PP cells were added to the Caco-2 cells; in an alternate technique, addition of human Raji B lymphoblastoid cells to Caco-2 cells also yielded epithelial monolayers with enhanced transcytotic function (24). Variations of the original Caco-2/Raji coculture model have been used widely to study transcytosis of nanoparticles and bacteria (13, 19, 33). A weakness of the Caco-2/Raji coculture model is that most of the genes selectively expressed by natural intestinal M cells are not induced in this in vitro model compared with monocultures of Caco-2 cells (31). There continues to be a need for additional in vitro models for study of intestinal M cells that use nontransformed cells and more faithfully replicate the transcriptional signature of the M cell lineage.

The enteroid culture system is a three-dimensional culture technique using a Matrigel scaffold with defined growth factors to enable the survival and expansion of stem cells present in freshly harvested intestinal crypts (48). Enteroids can be used to study the differentiation of specialized enterocytes found in the small intestinal epithelium by allowing maintenance of some intestinal stem cells (ISCs) in a reconstituted stem cell niche while permitting differentiation of some progeny of the precursor cells into specialized absorptive and secretory cell types naturally found in the intestinal epithelium (32). The enteroid system allows for the study of the intestinal epithelium without confounding signals from the microbiota and immune system, thus providing a physiologically relevant model for renewal and differentiation of the isolated intestinal epithelium. Addition of RANKL to mouse and human enteroid cultures was previously shown to induce M cell-associated gene expression and enhanced transcytosis of microspheres and bacteria (11, 40, 41). In the current study we have used the RANKL-supplemented enteroid culture system to further investigate signaling pathways involved in the differentiation of M cells. We find that RANKL acts through the noncanonical NF-κB pathway to induce *Spib* expression, followed by expression of Spi-B-dependent and -independent M cell-associated genes. We also show that while TNF-α alone does not induce M cell differentiation in enteroid cultures, TNF-α + RANKL boosts the expression of multiple M cell-associated genes compared with RANKL alone.

## MATERIALS AND METHODS

### 

#### Mice.

Female C57BL/6 mice (Jackson Laboratories, Bar Harbor, ME) were used for wild-type enteroid cultures. Alymphoplasia (*aly/aly*) mice and *aly/+* controls were bred in our mouse colony at Emory University starting with *aly/+* mice backcrossed onto the C57BL/6 background (provided by Drs. Mandy Ford and Kenneth Newell, Emory University). Mice were genotyped for the wild-type and *aly* mutant alleles of *Map3k14* (mitogen-activated protein kinase kinase kinase 14) gene by running two separate PCRs with allele-specific forward primers [5′-CACATCCCGAGCTACTTCAACA-3′ for *aly* and 5′-CACATCCCGAGCTACTTCAACG-3′ for wild-type NF-κB-inducing kinase (NIK)] and a common reverse primer (5′-CCTTCGGGGACTCTACAGGC-3′ for NIK) (50). The mutant and wild-type NIK alleles both yielded 266-bp PCR products. Mice with conditional deletion of the *Tnfrsf11a* gene encoding RANK in intestinal epithelial cells (RANK^ΔIEC^) and RANK^F/F^ littermate controls were bred at Emory University and genotyped as previously described (40). The animal studies were reviewed and approved by the Emory University Institutional Animal Care and Use Committee.

#### Crypt isolation and enteroid culture.

The distal 10 cm of the small intestine excluding any PPs was excised, opened, and washed. The intestine was then incubated with 5 mM EDTA for 20 min with shaking at 4°C. The epithelium was removed by manual disruption for 2 min in a solution of 43.4 mM sucrose (Thermo Fisher Scientific, Waltham, MA) and 54.9 mM d-sorbitol (Thermo Fisher Scientific) in Dulbecco's PBS (Corning Life Sciences, Tewksbury, MA). After filtration through 70-μm mesh and a 4-min 200-*g* spin, the sedimented crypts were resuspended in 50 μl of Matrigel (Corning Life Sciences) and placed in the center of the wells in a 24-well plate. The plates were incubated at 37°C for 30 min to allow for polymerization of Matrigel before addition of culture medium (500 μl/well) as previously described (32). The ENR culture medium (which includes EGF, Noggin, and R-spondin) consisted of 50:50 DMEM-Ham's F-12 (Corning Life Sciences), 1% N-2 Plus media supplement (R & D Systems, Minneapolis, MN), 2% B-27 serum-free supplement (Thermo Fisher Scientific), 1% penicillin-streptomycin (Corning Life Sciences), 10 mM HEPES (Thermo Fisher Scientific), 50 ng/ml EGF (Peprotech, Rocky Hill, NJ), 100 ng/ml Noggin (Peprotech), and 10% R-spondin2 conditioned medium obtained from the HEK-Rspo2AP cell line (provided by Dr. Jeffrey Whitsett, Cincinnati Children's Hospital Medical Center, Cincinnati, OH) (4). The ENR medium also contained the ROCK inhibitor Y-27632 (3 ng/ml; BD Biosciences, Franklin Lakes, NJ), which improved the viability of cultured enteroids. Newly established enteroids were cultured for 3 days before medium above the Matrigel was changed, and the cultures were stimulated with 100 ng/ml murine RANKL (Peprotech) for 1 or 3 days. In some experiments, enteroids were stimulated with 50 ng/ml murine IL-22 (Peprotech) or 50 ng/ml murine TNF-α (Peprotech) alone or in combination with RANKL. TNF-α was not used at >50 ng/ml, because higher concentrations led to increased enterocyte death due to apoptosis and compromised recovery of RNA (17).

#### Antibodies.

Ultra-LEAF-grade purified anti-mouse TNF-α antibody (MP6-XT22, BioLegend, San Diego, CA) was added to some enteroid cultures at a final concentration of 5 μg/ml to neutralize TNF-α. Unconjugated monoclonal rat anti-mouse GP2 (clone 2F11-C3, MBL International, Woburn, MA) was used to stain frozen sections of enteroids. Alexa Fluor 546-conjugated goat anti-rat secondary antibody (Invitrogen, Carlsbad, CA) was used to detect the anti-GP2 primary. FITC-conjugated monoclonal anti-E-cadherin antibody (clone 36, BD Biosciences) was used to stain cell junctions of enteroids on frozen sections.

#### Quantitative real-time PCR.

Enteroids in Matrigel were incubated with Cell Recovery Solution (Corning Life Sciences) with shaking for 1 h at 4°C to dissolve Matrigel prior to RNA extraction. The contents of three separate replicate wells were pooled for each experimental condition. After two PBS washes, RNA was extracted using the RNeasy Mini Kit (Qiagen, Valencia, CA). The iScript cDNA synthesis kit (Bio-Rad, Hercules, CA) was used to make DNA from 0.5–1.0 μg of RNA, with the same amount of RNA used from all samples in each experiment. iTaq Universal SYBR Green supermix (Bio-Rad) was used for PCRs run on a CFX Connect thermal cycler (Bio-Rad). All PCR primers are listed in Table 1. The amplicons from all primer pairs span at least one intron in the target gene to avoid amplification of genomic DNA. Each time a new primer pair was used for the first time, the size of the amplicon was confirmed on an agarose gel. Thereafter, the melting curves of the amplicons were used to confirm primer specificity. All amplifications were run in triplicate. Cycle threshold (C_t_) results were normalized by comparison with the housekeeping genes *Gapdh* and *Rpl13a* (ribosomal protein L13a). The ΔΔC_t_ method was used to determine fold induction of a gene of interest in a comparison of two samples, with normalization of each experimental C_t_ result to the geometric mean of the C_t_ values of *Gapdh* and *Rpl13a* (49). Expression of genes was reported relative to *Gapdh*, a widely used standard, and determined by normalizing the average C_t_ of each sample to the *Gapdh* result and setting the expression level of *Gapdh* at 1. The baseline expression of M cell-associated genes, including *Spib* and *Gp2*, in enteroids harvested 4 and 6 days after culture initiation was similar to the level of expression detected in freshly isolated small intestinal villous epithelium (data not shown).

**Table 1.** Primers for quantitative PCR

Name	5′-3′ Sequence	Source
Common *Spib* (forward)	GCCCACACTTAAGCTGTTTGTA	Sato et al. (43)
*Spib-1* (forward)	CTCTGAACCACCATGCTTGCT	Bartholdy et al. (3)
*Spib-2* (forward)	AGGGCGGCCCTGACAT	
Common *Spib* (reverse)	CTGTCCAGCCCCATGTAGAG	Sato et al. (43)
*Gp2*		
Forward	CTGCTACCTCGAAGGGGACT	qPrimerDepot (10)
Reverse	CATTGCCAGAGGGAAGAACT	
*Ccl9*		
Forward	TACTGCCCTCTCCTTCCTCA	Kanaya et al. (23)
Reverse	TTGAAAGCCCATGTGAAACA	
*Anxa5*		
Forward	ATCCTGAACCTGTTGACATCCC	PrimerBank (47)
Reverse	AGTCGTGAGGGCTTCATCATA	
*Ccl20*		
Forward	TCCAGAGCTATTGTGGGTTTCA	PrimerBank (47)
Reverse	GAGGAGGTTCACAGCCCTTTT	
*Tnfrsf11b*		
Forward	GGGCGTTACCTGGAGATCG	Akiyama et al. (1)
Reverse	GAGAAGAACCCATCTGGACATTT	
*Tnfaip2*		
Forward	TACTGCCCTCTCCTTCCTCA	qPrimerDepot (10)
Reverse	TTGAAAGCCCATGTGAAACA	
*Reg3g*		
Forward	CGTGCCTATGGCTCCTATTGCT	Natividad et al. (35)
Reverse	TTCAGCGCCACTGAGCACAGAC	
*Marcksl1*		
Forward	GGCAGCCAGAGCTCTAAGG	Sato et al. (43)
Reverse	TCACGTGGCCATTCTCCT	
*Saa1*		
Forward	CATTTGTTCACGAGGCTTTCC	Ivanov et al. (22)
Reverse	GTTTTTCCAGTTAGCTTCCTTCATGT	
*Relb*		
Forward	ACTGGATGCCCAGGTTGTTA	qPrimerDepot (10)
Reverse	CCTGGTGTGGAAGGACTGG	
*Nfkb2*		
Forward	GGCCGGAAGACCTATCCTACT	PrimerBank (47)
Reverse	CTACAGACACAGCGCACACT	
*Rpl13a*		
Forward	CACTCTGGAGGAGAAACGGAAGG	Cervia et al. (6)
Reverse	GCAGGCATGAGGCAAACAGTC	
*Gapdh*		
Forward	TTCACCACCATGGAGAAGGC	Larderet et al. (29)
Reverse	GGCATGGACTGTGGTCATGA	

#### Cloning of PCR-amplified Spib transcripts.

cDNA amplified from RANKL-treated enteroids using *Spib-1*- or *Spib-2*-specific primers was ligated into pJET1.2 using the CloneJET PCR cloning kit (Thermo Fisher Scientific). Plasmids with inserts of the correct size were sequenced by automated dideoxy sequencing. The sequences of the *Spib-1* and *Spib-2* amplicons confirmed use of the expected promoters and splice sites.

#### Immunofluorescence microscopy.

Enteroids were removed from Matrigel by treatment with 500 μl of Cell Recovery Solution (Corning Life Sciences) with shaking for 1 h at 4°C followed by washes. The recovered enteroids were embedded in optimum cutting temperature compound (OCT, Thermo Fisher Scientific) and snap-frozen in isopentane on dry ice. Blocks containing the enteroids were sectioned on a cryostat to yield 5-μm-thick frozen sections. Slides were fixed with 4% paraformaldehyde for 20 min at room temperature. Sections were stained overnight with rat anti-mouse GP2 monoclonal antibody and then incubated for 2 h with goat anti-rat IgG-Alexa Fluor 546 secondary antibody and monoclonal FITC-anti-E-cadherin antibody. 4′,6-Diamidino-2-phenylindole (EMD Millipore, Billerica, MA) was used to stain nuclei. Fluorescence staining images were acquired with a Nikon 50i microscope using an ×40 oil-immersion objective.

#### Statistical analysis.

Mean values of relative expression in quantitative PCR experiments were compared by a two-tailed Student's *t*-test with Prism (GraphPad Software, La Jolla, CA). *P* < 0.05 was considered significant.

## RESULTS

### 

#### Enteroids stimulated with RANKL express M cell-associated genes.

**Fig. 1.**
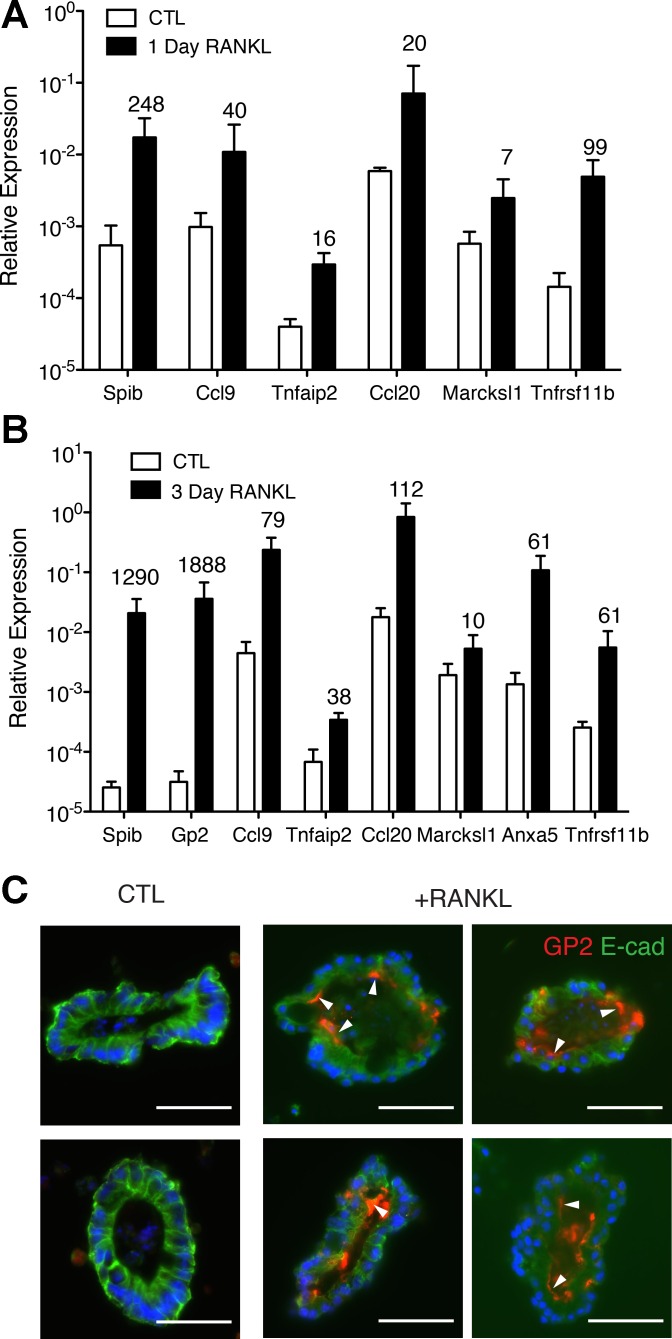
Enteroids stimulated with receptor activator of NF-κB ligand (RANKL) express M cell-associated genes. *A* and *B*: enteroids were stimulated with 100 ng/ml RANKL for 1 or 3 days. Expression of genes was determined by quantitative PCR and reported as relative expression normalized to *Gapdh*. Numbers above bars indicate fold induction of M cell-associated genes compared with untreated controls. Values are means ± SE of 4 experiments. Relative expression of all genes examined was increased after RANKL treatment compared with controls (*P* < 0.05 for each gene). *Ccl9* and *Ccl20*, chemokine (C-C motif) ligands 9 and 20; *Tnfaip2*, TNF-α-induced protein 2; *Marcksl1*, myristoylated alanine-rich protein kinase C substrate; *Anxa5*, annexin A5; *Tnfrsf11a*, TNF-α receptor superfamily member 11A; *Gp2*, glycoprotein 2. *C*: immunofluorescence of control enteroids and enteroids treated with 100 ng/ml RANKL for 3 days. Blue, 4′,6-diamidino-2-phenylindole; green, E-cadherin (E-cad); red, GP2. Arrowheads indicate apical GP2 staining on single cells. CTL, control. Scale bars = 50 μm.

Three-dimensional enteroid cultures were established using C57BL/6 small intestinal crypts and cultured for 3 days in ENR medium. The medium was replaced at 3 days with ENR or ENR supplemented with RANKL to induce expression of M cell-associated genes. At 1 day after RANKL addition, mRNA for several genes known to be selectively expressed by M cells and/or the FAE, including *Spib*, *Ccl9*, *Tnfaip2* (TNF-α-induced protein 2), *Marcksl1*, and *Ccl20*, was strongly upregulated (Fig. 1*A*). Induction of *Ccl9* and *Tnfaip2* in M cells was previously shown to be Spi-B-dependent, while induction of *Marcksl1* is independent of Spi-B (23). Another gene strongly induced by RANKL at 1 day was *Tnfrsf11b*, which encodes osteoprotegerin, a soluble decoy receptor for RANKL that functions as an antagonist of RANKL-mediated signaling. Induction of *Tnfrsf11b* by RANKL was previously demonstrated in thymic medullary epithelial cells (1). After 3 days of stimulation with RANKL, additional M cell-associated genes expressed by mature M cells, including *Gp2* and *Anxa5*, were induced (Fig. 1*B*). To ascertain the frequency of M cell differentiation within enteroids after addition of RANKL, sections of enteroids stimulated with RANKL for 3 days were stained for GP2 (Fig. 1*C*). GP2 was detected predominantly on the apical surface of an average of three to four cells per RANKL-stimulated enteroid, and 11% of the total cells were examined. No GP2 expression was detected in control cultures that did not receive RANKL. The GP2^+^ M cells within each enteroid were usually not found adjacent to other M cells, which resembles the pattern of distribution of M cells within the FAE of PPs. The M cell-associated genes induced by RANKL in vitro in enteroid cultures were expressed in the same sequence previously described in vivo for the small intestinal villous epithelium following systemic RANKL injection (23).

#### RANKL, IL-22, and TNF-α induce distinct patterns of gene expression in enteroids.

**Fig. 2.**
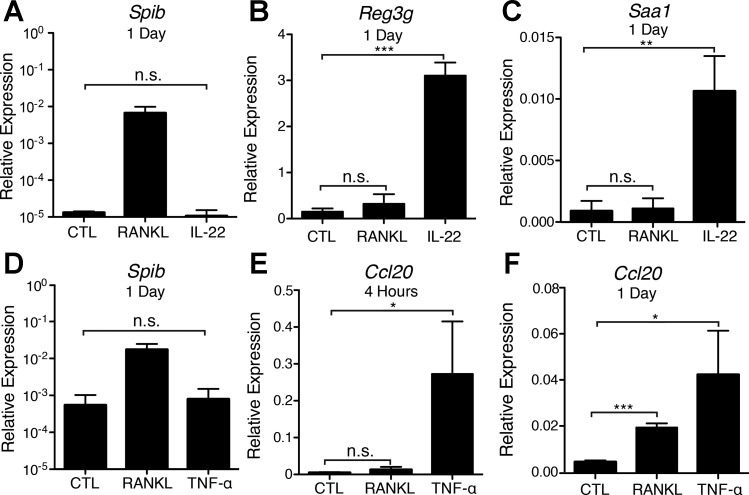
RANKL specifically induces *Spib* in enteroids. *A–C*: relative expression of *Spib*, *Reg3g* (regenerating islet-derived protein 3γ), and *Saa1* (serum amyloid A1) in untreated enteroids and enteroids treated with RANKL (100 ng/ml) or IL-22 (50 ng/ml) for 1 day. Values are means ± SE of 3 experiments. *D–F*: relative expression of *Spib* and *Ccl20* in untreated enteroids or enteroids treated with RANKL (100 ng/ml) or TNF-α (50 ng/ml) at 1 day and 4 h. Values are means ± SE of 4 experiments. *P < 0.05; **P < 0.01; ***P < 0.001. ns, Not significant.

IL-22 has potent effects on intestinal epithelial cells in vivo and in enteroid cultures, signaling through a heterodimeric receptor consisting of IL-22R1 and IL-10R2 to activate STAT3 and induce epithelial proliferation and expression of antimicrobial proteins (30, 34). To demonstrate the specificity of RANKL-induced gene expression in the enteroid system, cultures were stimulated for 1 day with IL-22 or RANKL or maintained in the base ENR medium. Addition of IL-22 strongly induced known IL-22 responsive genes, including *Reg3g* (regenerating islet-derived protein 3γ) and *Saa1* (serum amyloid A1), but did not induce *Spib* (Fig. 2, *A–C*). No induction of *Reg3g* and *Saa1* was observed after RANKL stimulation under conditions that resulted in strong induction of *Spib*. TNF-α is a cytokine in the TNF superfamily that rapidly activates the canonical, but not the slower noncanonical, NF-κB pathway (14, 18, 36). Stimulation of enteroids with TNF-α induced *Ccl20* expression at 4 h and 1 day but failed to induce *Spib* expression at 1 day (Fig. 2, *D–F*). RANKL also induced *Ccl20* expression, but not until the 1-day time point (Fig. 2, *E* and *F*). While the enteroid cultures responded to all three cytokines tested, only RANKL induced an increase in *Spib* expression.

#### RANKL-induced M cell differentiation depends on the noncanonical NF-κB signaling pathway.

**Fig. 3.**
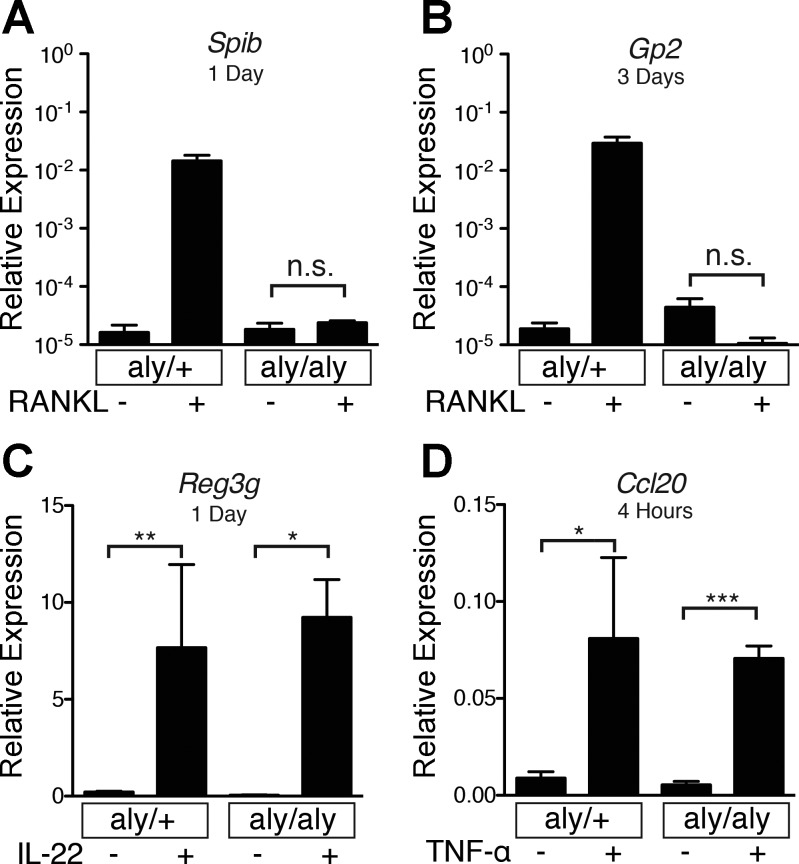
*Aly/aly* enteroids do not express M cell-specific genes when stimulated with RANKL. *A* and *B*: relative expression of *Spib* and *Gp2* following stimulation with RANKL (100 ng/ml) for 3 days in Alymphoplasia control (*aly/+*) and *aly/aly* enteroids. Values are means ± SE of 3 experiments. *C*: relative expression of *Reg3g* following treatment with IL-22 (50 ng/ml) for 4 h in *aly/+* and *aly/aly* enteroids. Values are means ± SE of 3 experiments. *D*: relative expression of *Ccl20* following treatment of *aly/+* and *aly/aly* enteroids with TNF-α (50 ng/ml) for 1 day. Values are means ± SE of 2 experiments. *P < 0.05; **P < 0.01; ***P < 0.001. ns, Not significant.

RANK is one of several receptors in the TNF receptor superfamily that signals primarily through the noncanonical NF-κB signaling pathway, which depends on activation of NIK and nuclear translocation of p52-RelB heterodimers (8, 9, 12, 36). To determine if RANKL-induced M cell differentiation in enteroids also requires the noncanonical NF-κB pathway, enteroids from *aly/aly* mice with a nonfunctional NIK allele and *aly/+* control mice were stimulated with RANKL, IL-22, or TNF-α (Fig. 3). RANKL failed to induce *Spib* or *Gp2* expression in *aly/aly* enteroids. However, *aly/aly* and *aly/+* enteroids showed nearly equivalent induction of *Reg3g* by IL-22. In addition, TNF-α induction of *Ccl20* via the canonical NF-κB pathway was maintained in *aly/aly* enteroids (Fig. 3*D*). These results show that RANKL-induced M cell differentiation in enteroids is abrogated when noncanonical NF-κB signaling is blocked.

#### RANKL induces the NF-κB-dependent Spib-1 transcript variant.

**Fig. 4.**
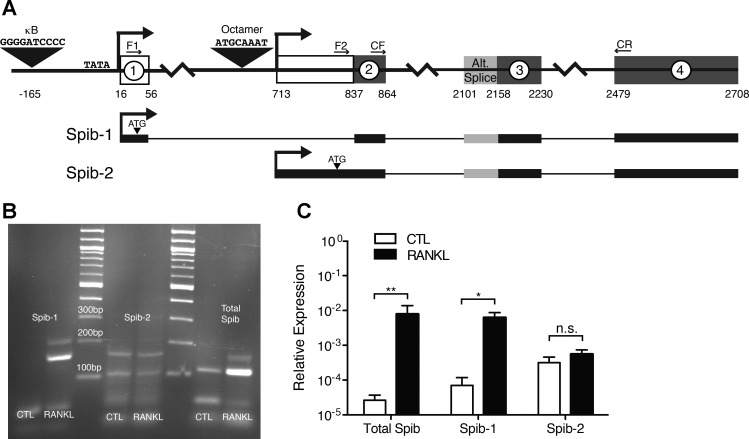
RANKL stimulation of enteroids induces the *Spib-1* transcript of *Spib*. *A*: schematic of mouse chromosome 7 genomic DNA containing the promoters and first 4 exons of *Spib*. Exon-intron boundaries for the first 4 exons are numbered based on alignment of the National Center for Biotechnology Information Reference Sequence mRNA for mouse *Spib* (NM_019866.1) with 6,092 bp of C57BL/6 genomic chromosome 7 sequence (NC_000073.6, 44525995..44532086, complement), with nucleotide position 1 assigned based on the predicted 5′ end of a *Spib* mRNA with the maximum amount of 5′-untranslated sequence. Large arrowheads indicate κB and octamer binding sites located in the 1st and 2nd promoters, respectively. Individual exons are indicated by numbers in white circles. Exons or regions of exons in white boxes are unique to the *Spib-1* and *Spib-2* transcripts transcribed from promoters 1 and 2, respectively; exons in dark-gray boxes are present in both *Spib-1* and *Spib-2*. Light gray portion at the 5′ end of exon 3 is an alternatively spliced region included in some *Spib* splice variants. F1, *Spib-1*-specific forward primer; F2, *Spib-2*-specific forward primer; CF, *Spib* common forward primer; CR, common reverse primer. *B*: agarose gel of PCR products obtained with *Spib-1*-specific, *Spib-2*-specific, and common *Spib* forward primers using cDNA from control enteroids and enteroids stimulated with 100 ng/ml RANKL for 1 day. *C*: relative expression of total *Spib*, *Spib-1*, and *Spib-2* after 1 day of stimulation with 100 ng/ml RANKL. Values are means ± SE of 3 experiments. *P < 0.05; **P < 0.01. ns, Not significant.

In B lymphocyte cell lines, transcription of the mouse *Spib* gene can be initiated from two distinct promoters, yielding transcript variants designated *Spib-1* and *Spib-2* (1, 3, 7). The promoter regulating expression of the *Spib-1* transcript is located upstream of the first exon, while the promoter of the *Spib-2* transcript is found within the first intron (Fig. 4*A*). The *Spib-1* promoter contains a consensus κB site (GGGGATCCCC) 149 bp upstream of a consensus TATA box sequence (TATATATA) located just 5′ to the transcriptional start site. The *Spib-2* promoter includes a recognition site for octamer transcription factors (ATTTGCAT) but does not include a TATA box (3, 7). Because the primer pair used in our previous quantitative PCR experiments amplifies the *Spib-1* and *Spib-2* transcripts, we used isoform-specific forward primers in combination with a common reverse primer to determine which *Spib* transcript was induced by RANKL in enterocytes (Fig. 4, *B* and *C*). The isoform-specific quantitative PCR amplifications revealed that RANKL exclusively induced the *Spib-1* transcript. A low constitutive level of *Spib-2* mRNA was detected in enteroids, but no additional *Spib-2* mRNA was induced by addition of RANKL (Fig. 4*C*). The previously reported splice junctions between the first and second exons of the *Spib-1* and *Spib-2* transcripts were confirmed by cloning and sequencing the isoform-specific *Spib-1* and *Spib-2* amplicons (M. B. Wood, data not shown). Thus, RANKL-induced *Spib* expression in enterocytes depends on noncanonical NF-κB induction of the *Spib-1* transcript.

#### TNF-α enhances RANKL-induced M cell-associated gene expression.

**Fig. 5.**
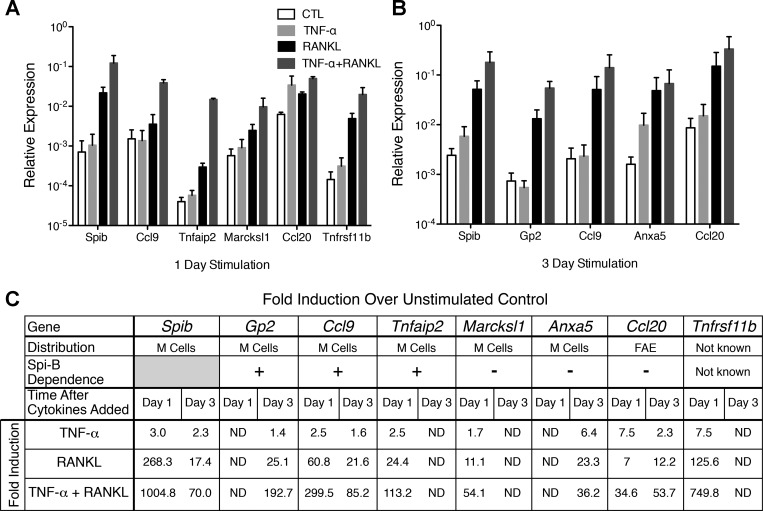
TNF-α enhances RANKL-induced M cell-associated gene expression. *A*: relative expression of M cell-specific and follicle-associated epithelium (FAE)-specific genes after no stimulation or 1 day of stimulation with 50 ng/ml TNF-α, 100 ng/ml RANKL, or 50 ng/ml TNF-α + 100 ng/ml RANKL. Values are means ± SE of 3 experiments. Expression of all genes was increased in TNF-α + RANKL- compared with RANKL-treated enteroids (*P* < 0.05). *B*: relative expression of M cell-specific genes after 3 days of stimulation with 50 ng/ml TNF-α, 100 ng/ml RANKL, or 50 ng/ml TNF-α + 100 ng/ml RANKL. Values are means ± SE of 3 experiments. Expression of all genes except *Anxa5* was significantly increased in TNF-α + RANKL- compared with RANKL-treated enteroids (*P* < 0.05). *C*: average fold induction of M cell- and FAE-specific genes by RANKL and TNF-α + RANKL. ND, not determined.

**Fig. 6.**
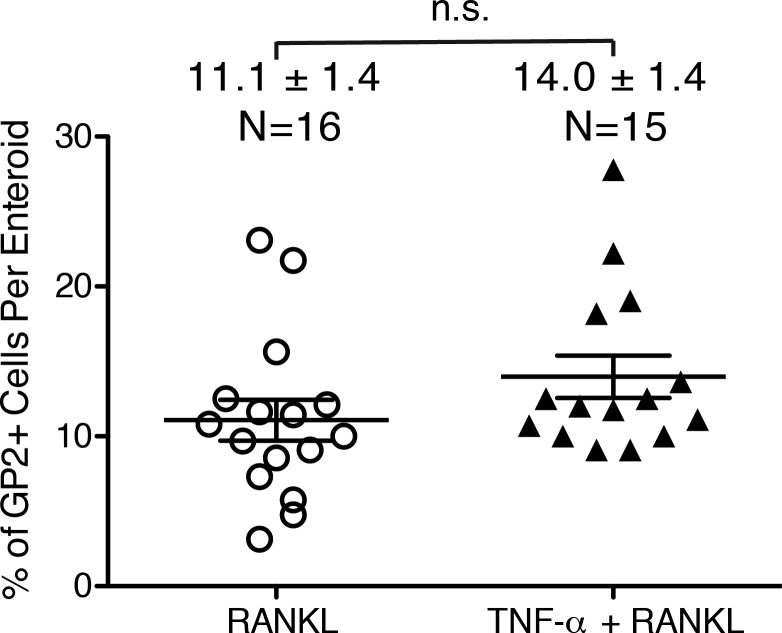
Addition of TNF-α does not increase the frequency of GP2^+^ M cells in RANKL-treated enteroids. GP2^+^ cells as a percentage of the total number of DAPI^+^ nuclei was compared in sections of enteroids treated for 3 days with 100 ng/ml RANKL or 50 ng/ml TNF-α + 100 ng/ml RANKL. Results are presented as a scatter plot of data collected from individual enteroids. Means ± SE for each group and number of enteroids are shown above plot. ns, Not significant.

TNF-α signaling through the canonical NF-κB pathway can synergize with RANKL in the in vitro induction of genes associated with osteoclast differentiation (16, 27, 51). TNF-α also plays an essential, supportive role in the normal maturation of B cell follicles in lymphoid tissues, including PPs (28, 38, 39). To determine whether TNF-α can also support RANKL-induced M cell differentiation, enteroid cultures were stimulated with RANKL, TNF-α, or TNF-α + RANKL. While stimulation with TNF-α alone resulted in little, if any, increase in expression of the M cell-associated genes, TNF-α + RANKL consistently resulted in a three- to sixfold boost in expression of the M cell-associated genes over the considerable induction achieved with RANKL alone (Fig. 5). This robust increase in expression of M cell-associated genes after stimulation with TNF-α + RANKL was associated with only a small increase in the frequency of GP2^+^ cells in enteroids (to 14% compared with 11% with RANKL only) that was not statistically significant (Fig. 6). These results indicate that the enhancement of M cell-associated gene expression by stimulation with TNF-α + RANKL is primarily achieved by more rapid induction of M cell-associated gene expression, rather than recruitment of additional precursor cells into the M cell lineage.

#### TNF-α fails to induce M cell-specific gene expression in the absence of RANKL-RANK signaling.

**Fig. 7.**
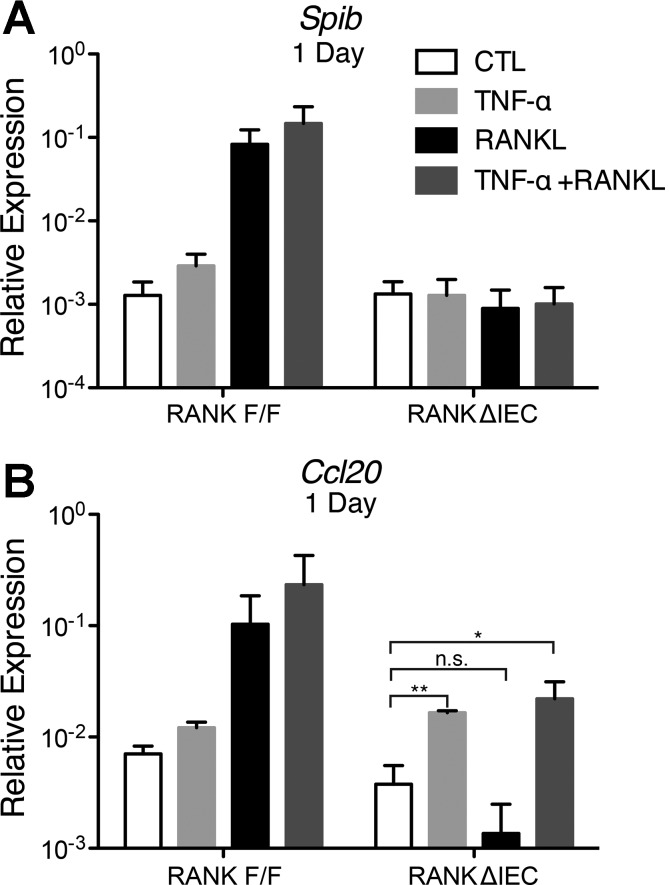
TNF-α does not induce *Spib* expression in RANK^ΔIEC^ enteroids. *A* and *B*: relative expression of *Spib* and *Ccl20* by enteroids from mice with conditional deletion of the *Tnfrsf11a* gene encoding RANK in intestinal epithelial cells (RANK^ΔIEC^) and RANK^F/F^ littermate controls that received no stimulation or enteroids stimulated with 50 ng/ml TNF-α, 100 ng/ml RANKL, or 50 ng/ml TNF-α + 100 ng/ml RANKL. Values are means ± SE of 3 experiments. *P < 0.05; **P < 0.01; ns, Not significant.

A slight induction of *Spib* and several Spi-B-dependent genes was seen in some TNF-α stimulation experiments using C57BL/6 enteroids, perhaps as a result of TNF-α enhancing the response to a small amount of residual endogenous RANKL in the enteroids. This low and variable level of induction of M cell-associated genes by TNF-α alone was not detected in enteroids cultured from RANK^ΔIEC^ mice, in which any endogenous RANKL would be unable to signal (Fig. 7). TNF-α was able to normally induce *Ccl20* expression in enteroids from RANK^ΔIEC^ mice, confirming that the canonical NF-κB pathway remained intact.

#### TNF-α stimulates transcription of Relb and Nfkb2 in enterocytes.

**Fig. 8.**
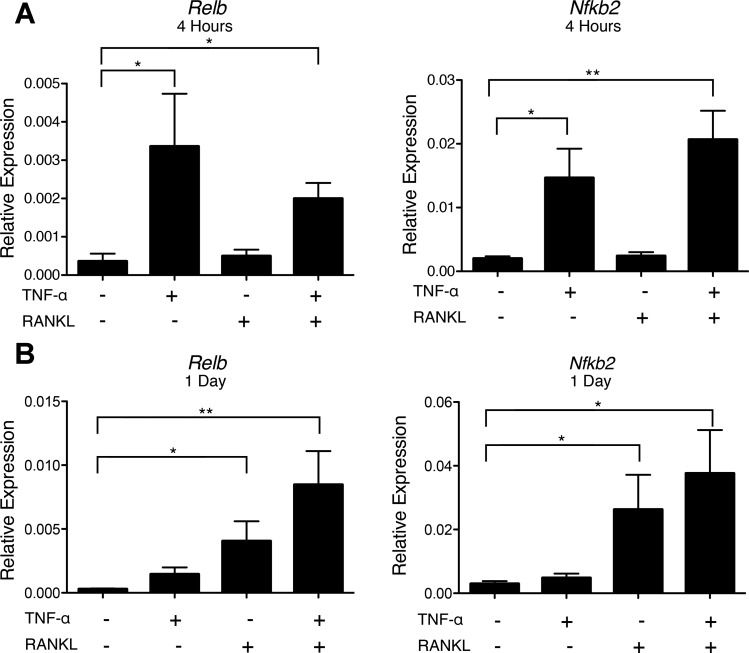
TNF-α induces *Relb* and *Nfkb2* expression and enhances RANKL-induced *Relb* and *Nfkb2* (NF-κB subunit 2). *A*: relative expression of *Relb* and *Nfkb2* after 4 h of no stimulation or stimulation with 50 ng/ml TNF-α, 100 ng/ml RANKL, or 50 ng/ml TNF-α + 100 ng/ml RANKL. Values are means ± SE of 3 experiments. *B*: relative expression of *Relb* and *Nfkb2* after 1 day of no stimulation or stimulation with 50 ng/ml TNF-α, 100 ng/ml RANKL, or 50 ng/ml TNF-α + 100 ng/ml RANKL. Values are means ± SE of 4 experiments. *P < 0.05; **P < 0.01.

TNF-α signaling through the canonical NF-κB pathway is known to induce transcription of *Relb* in several cell types (5, 53). We determined whether TNF-α stimulation of enterocytes induced the genes encoding the RelB and p52 components of the noncanonical NF-κB heterodimer. After 4 h of stimulation with TNF-α, expression of *Relb* and *Nfkb2* was significantly increased (Fig. 8). While stimulation with RANKL did not induce increases in *Relb* or *Nfkb2* at 4 h, enteroids treated for 1 day with RANKL showed significant induction of *Relb* and *Nfkb2* expression. TNF-α + RANKL resulted in more induction of *Relb* and *Nfkb2* at 1 day than RANKL alone. The early induction of *Relb* and *Nfkb2* by TNF-α results in greater availability of the two proteins that comprise the noncanonical NF-κB heterodimer that translocates to the nucleus after RANKL-induced activation of NIK.

#### RANKL-induced M cell-associated gene expression does not depend on endogenous TNF-α.

**Fig. 9.**
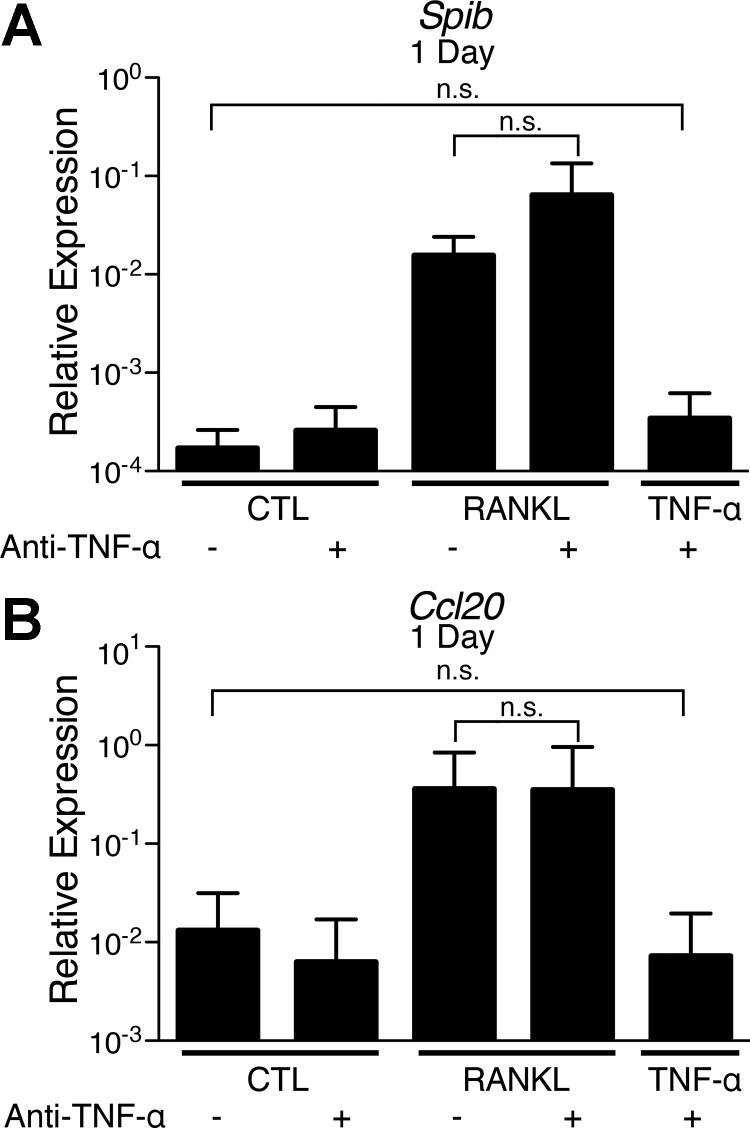
RANKL induces M cell-specific gene expression in the absence of endogenous TNF-α. *A* and *B*: relative expression of *Spib* and *Ccl20* by enteroids after 1 day of no stimulation (CTL) or stimulation with 50 ng/ml TNF-α or 100 ng/ml RANKL with or without a neutralizing anti-TNF-α antibody (5 μg/ml). Values are means ± SE of 3 experiments. ns, Not significant.

The enhancing effect of TNF-α on RANKL-induced M cell differentiation raised the possibility of small amounts of endogenous TNF-α in enteroids, partially supporting the effects of RANKL. To test this possibility, we stimulated enteroid cultures with RANKL in the presence of a TNF-α-neutralizing antibody (Fig. 9). Anti-TNF-α did not reduce the RANKL-induced expression of *Spib* or *Ccl20*. The neutralizing activity of the anti-TNF-α in the enteroid system was confirmed by its ability to reduce expression of *Ccl20* in TNF-α-treated enteroids to the level of the untreated control. Thus, RANKL-induced M cell differentiation in the enteroid culture system is independent of endogenous TNF-α.

## DISCUSSION

The epithelial lining of the mammalian intestine consists of multiple specialized types of enterocytes that arise following differentiation of ISCs residing near the base of intestinal crypts. The identification of a defined, serum-free medium capable of supporting the in vitro growth of isolated ISCs in a three-dimensional matrix (“organoid” cultures) was an important technical advance in stem cell and epithelial cell biology (32, 44, 45). In vitro studies with enteroids (i.e., organoids established with small ISCs) are providing new insights into how ISCs differentiate into various specialized absorptive and secretory lineages (48). Antigen-sampling M cells belong to a highly specialized enterocyte lineage that is normally restricted to the FAE overlying gut-associated lymphoid tissue structures. While M cells are not detected in standard enteroid cultures, previous work has shown that RANKL supplementation of mouse and human enteroids is sufficient to elicit the appearance of a subset of cells expressing signature M cell genes (e.g., *Spib* and *Gp2*) and capable of enhanced phagocytic activity (11, 40, 41).

Because expression of *Spib* and *Gp2* is strongly induced by RANKL in the mouse enteroids, we investigated which other known M cell-associated genes were activated during the course of in vitro M cell differentiation. We found that Spi-B-dependent (*Ccl9* and *Tnfaip2*) and -independent (*Marcksl1* and *Anxa5*) M cell-specific genes were efficiently induced by RANKL. Because M cells found in RANKL-supplemented enteroids faithfully replicate the pattern of gene expression of natural M cells, the enteroid system is a significant improvement over the Caco-2/Raji coculture system for gene discovery applications and functional studies of bona fide M cells (31). The RANKL-supplemented enteroid system provides a new discovery tool for identification of novel M cell lineage-restricted genes in multiple species and can also be used to determine whether putative M cell-associated genes identified by other approaches are part of the RANKL-activated differentiation program.

Absorptive enterocytes within the FAE exhibit a pattern of gene expression different from that of absorptive enterocytes found on villi (21), and CCL20 is one of the best-characterized markers selectively expressed by FAE enterocytes (2, 52). RANKL induced *Ccl20* expression in enteroid cultures, indicating that RANKL is one of the endogenous signals contributing to the specific gene expression pattern characteristic of FAE enterocytes. Since other cytokines, including IL-1, TNF-α, and LTα_1_β_2_, also strongly induce *Ccl20* expression by enterocytes (14, 42), the combined effects of RANKL and additional cytokines present in the local PP microenvironment are likely to be responsible for the pattern of gene expression characteristic of FAE enterocytes. RANKL stimulation of thymic epithelial cells activates expression of the *Tnfrsf11b* gene encoding the soluble RANKL decoy receptor osteoprotegerin (1); therefore, we tested whether RANKL also induced *Tnfrsf11b* in enterocytes. RANKL strongly induced *Tnfrsf11b* in enterocytes, which may be part of an inhibitory feedback loop that regulates the enterocyte response to RANKL. We have not determined whether the RANKL-induced expression of *Tnfrsf11b* occurs throughout the FAE or only in M cells.

We also used the mouse enteroid system to investigate the relative roles of the canonical and noncanonical NF-κB signaling pathways in RANKL-induced M cell differentiation. RANKL failed to induce the M cell-specific genes *Spib* and *Gp2* in enteroid cultures established from *aly/aly* mice homozygous for a null mutation in the *Map3k14* gene encoding NIK, extending previous in vivo results obtained after injection of RANKL into *aly/aly* mice (25). The block of M cell differentiation in *aly/aly* mice shows that M cell differentiation has the same dependence on the noncanonical NF-κB pathway as most other RANKL-dependent responses, including the induction of *Spib* in mouse thymic epithelial cells (1). One of the important early targets for the noncanonical NF-κB heterodimer induced following RANKL stimulation is likely to be the κB site in the *Spib-P1* promoter upstream of the first exon of the mouse *Spib* gene. Addition of RANKL to enteroids induced the *Spib-1* mRNA isoform transcribed from this promoter, rather than the *Spib-2* mRNA transcribed from the *Spib-P2* promoter located upstream of the second exon. *Spib-P1* was previously shown to be the *Spib* promoter activated by RANKL in thymic epithelial cells (1).

Experiments comparing the responses of enteroid cultures with RANKL alone or TNF-α + RANKL demonstrated that TNF-α + RANKL consistently resulted in a three- to sixfold boost in the expression of M cell-associated genes above the level achieved with RANKL alone. Since TNF-α signals through the canonical, and not the noncanonical, NF-κB pathway (37), this result indicates that strong activation of the canonical NF-κB pathway can play a supporting role in RANKL-stimulated M cell differentiation. One potential mechanism for this effect is the ability of canonical NF-κB activation to rapidly induce enhanced expression of the *Relb* and *Nfkb2* genes encoding the RelB and p52 subunits of the noncanonical NF-κB heterodimer (46, 53). After RANKL-RANK signaling activates NIK, allowing for the p100 precursor protein to be processed into the active p52 subunit and to associate with RelB, the presence of more RelB and p100 protein increases the number of potential noncanonical NF-κB heterodimers that can form to mediate the downstream effects of RANKL-dependent NIK activation. Alternatively, some of the κB sites that regulate transcription of genes involved in M cell and FAE differentiation may be responsive to binding of canonical p65–p50 or noncanonical RelB-p52 heterodimers, potentially enabling synergistic gene induction when nuclear translocation of canonical and noncanonical NF-κB complexes occurs at the same time.

Our finding that TNF-α enhances RANKL-induced expression of the full spectrum of M cell-associated genes in enteroids has several implications for future studies of M cell differentiation. Stimulation of enteroids with TNF-α + RANKL may assist in the discovery of additional M cell-associated genes by enhancing the sensitivity of transcriptomics to detect genes that are less strongly induced. The supportive role of TNF-α demonstrated in vitro also raises the possibility that TNF-α, or even other inducers of the canonical NF-κB pathway in the PP microenvironment, could serve a similar function during in vivo M cell differentiation. However, our studies clearly show that a cytokine such as TNF-α, which can play an accessory role in M cell differentiation through activation of the canonical NF-κB pathway, is not capable of inducing *Spib* and the rest of the Spi-B-dependent M cell differentiation program on its own.

## GRANTS

This work was supported by National Institutes of Health Grants R01 DK-64730 and R21 AI-111388 (to I. R. Williams) and a Senior Research Award from the Crohn's & Colitis Foundation of America (to I. R. Williams).

## DISCLOSURES

No conflicts of interest, financial or otherwise, are declared by the authors.

## AUTHOR CONTRIBUTIONS

M.B.W. and I.R.W. developed the concept and designed the research; M.B.W. and D.R. performed the experiments; M.B.W., D.R., and I.R.W. analyzed the data; M.B.W., D.R., and I.R.W. interpreted the results of the experiments; M.B.W. prepared the figures; M.B.W. drafted the manuscript; M.B.W. and I.R.W. edited and revised the manuscript; M.B.W. and I.R.W. approved the final version of the manuscript.
